# Utilization of Drug-Coated Balloons for the Treatment of Coronary Lesions in the Elderly Population

**DOI:** 10.3390/jcm11092616

**Published:** 2022-05-06

**Authors:** Gal Sella, Gera Gandelman, Ortal Tuvali, Igor Volodarsky, Valeri Cuciuc, Dan Haberman, Omar Ayyad, Lion Poles, Michael Welt, Oscar Horacio Kracoff, Jacob George

**Affiliations:** The Heart Center, Kaplan Medical Center, The Hebrew University of Jerusalem, 1 Pasternak St., Rehovot 7661041, Israel; galse1@clalit.org.il (G.S.); ortalhaga@clalit.org.il (O.T.); igorv@clalit.org.il (I.V.); valeryku@clalit.org.il (V.C.); danha1@clalit.org.il (D.H.); omeray@clalit.org.il (O.A.); lion_p@clalit.org.il (L.P.); michaelwel@clalit.org.il (M.W.); oscar_k@clalit.org.il (O.H.K.); kobige@clalit.org.il (J.G.)

**Keywords:** revascularization, TLR, geriatrics, DCB, re-stenosis, atherosclerosis

## Abstract

Introduction: The use of drug-coated balloons (DCBs) has become more prevalent in the past few years for the treatment of in-stent restenosis (ISR) and de novo lesions. The absence of foreign polymer implantations potentially shortens the duration of dual anti-platelet therapy (DAPT), which can be beneficial for the elderly population. We aimed to investigate the safety and efficacy of the use of DCBs for the treatment of coronary lesions in elderly patients as compared to the younger population. Materials and methods: A database of 446 consecutive patients who underwent a procedure of DCB inflation in our institution was divided into two groups, below 70 years old and above 80 years old. We compared and analyzed the endpoints of total major adverse cardiovascular events (MACE), cardiovascular (CV) death, and all-cause mortality in both groups. Results: The difference in MACE between the two age groups was non-significant (*p* = 0.225); the difference in cardiovascular death was also non-significant (*p* = 0.086). All-cause mortality was significantly different (*p* < 0.0001) and can be attributed to the age of the patients. Conclusion: The utilization of DCBs for the treatment of coronary lesions may be as safe and effective for the elderly population as for the younger population and may allow a shorter period of DAPT therapy, which can lower the risk of bleeding.

## 1. Introduction

Drug-coated balloons have come to be more commonly used in the past few years. This novel revascularization method uses a semi-compliant balloon coated with the antiproliferative agent Paclitaxel to mainly treat in-stent restenosis lesions and off-label de novo coronary lesions of small vessels [1]. The inflation of a balloon distributing a locally active drug to treat coronary lesions is considered as safe and effective as the deployment of a polymer or metal stent in the diseased area. The formation of the scar tissue known as neointima within deployed stents gives rise to accelerated neoatherosclerosis and eventually a re-stenosis of the stent [2]. The inflation of the balloon itself after proper angioplastic preparation squashes the atherosclerotic plaque while the Paclitaxel elution mainly prevents the growth of neointima within re-stenosed stents, thus making them less likely to re-stenose again in the future [3].

The efficacy of the use of DCB for treating de novo lesions has been increasingly studied in recent years [4]. Although showing mixed results, and despite being used “off-label”, DCB has become part of the arsenal routinely used in many catheterization labs as an alternative for DES deployment in the treatment of de novo lesions in small vessels [5].

Frailty is a geriatric syndrome consisting of increased vulnerability to stressors that have been involved as a causative and prognostic factor in patients with cardiovascular disease (CVD) [6]. This syndrome and its consequences have increasingly been studied in the past two decades. Recent studies show a close connection between atherosclerotic burden and increased risk of frailty [7]. Moreover, frailty has recently been shown to be associated with an increased susceptibility to cardiovascular disease [8]. This reciprocal relationship requires the early and accurate treatment of its confounders, especially CVD [9], in order to slow down the progression of frailty and avoid further disabilities.

As life expectancy increases, awareness regarding optimal, custom-made, and patient-centered medical treatment for the third age and their special needs is rising [10]. Drug dosing adjusted to age groups, task forces on geriatric cardiology, and novel treatment approaches [11,12,13] are all consequences of recent studies emphasizing the importance of considering the aging society as a special population; this recognition inclines the scientific community to seek to reshape existing modalities to suit the elderly.

The overall safety profile, the efficacy, and the factor of not “leaving behind” a potentially thrombogenic substance are making DCB a promising alternative to DES in the elderly population [14].

In this study, we aim to investigate the mid-term outcomes of DCBs in geriatric patients compared to younger (below 70-years old) patients; the rationale is to establish new insights based on our own experience which can encourage further work-ups and may even be incorporated in guidelines in the future.

## 2. Methods

### 2.1. Patients’ Population, Inclusion and Exclusion Criteria

The study protocol was reviewed and approved by our institutional ethics committee (Helsinki Committee for Human Rights—Kaplan Medical Center, Rehovot, Israel) before the study began. All data were fully anonymized before access and the ethics committee waived the requirement for informed consent.

**Figure 1:**
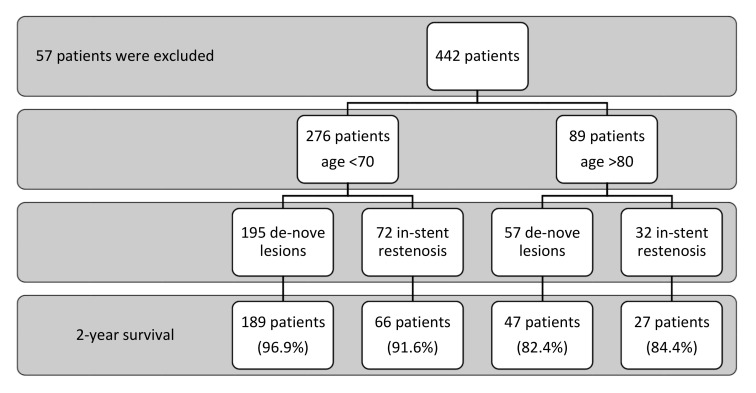
Patient population.

All consecutive patients in our institution who underwent DCB were retrospectively included (total 442 patients). A division into two cohorts was performed: 80 years old and above (elderly cohort); and 70 years old and below (young cohort). Patients aged between 71 and 79 were excluded in order to avoid a “gray zone”. Eligible patients were those with lesions in the coronary vessel tree who were planned to be treated with DCB only, as directed by the interventional cardiologist (Figure 1).

All lesion locations and characteristics were included. Bailout-stented lesions due to severe dissection after DEB were included. Lesions pre-specified to be stented (e.g., bifurcations) were excluded. Other exclusion criteria were procedures that included a mixture of DEB and stent implantation in different sites and stage PCI patients with an interval of two months.

### 2.2. Procedure Description

Femoral or radial access was allowed at the operator’s discretion with introducer sheaths of at least 5 French. During the intervention, close adherence to the recommendations of the ESC guidelines on how to use the DCBs in coronary artery disease was strongly endorsed. Special emphasis was paid to an adequate lesion preparation prior to DCB treatment; predilatation with uncoated balloons with a balloon-to-vessel ratio of 0.8–1.0 and inflation pressures exceeding a nominal pressure were mandatory. Second generation paclitaxel-iopromide DCB angioplasty with a SeQuent Please^®^ (B. braun, Melsungen, Germany) or a Pantera LUX^®^ (Biotronik, Berlin, Germany) balloon catheter was performed in the absence of a major flow-limiting dissection. Periprocedural and post-procedure medication protocols included the intravenous administration of heparin (70 IU/kg); the lifelong administration of acetylsalicylic acid (ASA) at 75–325 mg/day, and a clopidogrel loading dose of at least 300 mg, complemented with a regimen of 75 mg/day for 12 months; four weeks of clopidogrel therapy was recommended for high bleeding risk (HBR) patients [15]. In the acute coronary syndrome (ACS) setting, 12 months of dual-antiplatelet treatment was recommended [15]. Prasugrel or ticagrelor could be used instead of clopidogrel based on applicable guidelines [15], with their loading occurring at the end of the procedure.

### 2.3. Study Definition Criteria for Events

The primary endpoint was a clinically driven target lesion revascularization rate (TLR) at 24 months. Secondary endpoints were the mortality rate at 24 months; major adverse cardiac events defined as the composite of TLR; the number of cardiac hospitalizations, including myocardial infarctions (MI); and cardiac death and definite vessel thrombosis after 24 months of follow-up. MI was defined according the Universal Definition Guidelines [16]. Definite acute/subacute vessel thrombosis was defined according to the ARC criteria [17]. When the cause of death was unknown (e.g., sudden death at home), it was assumed to be of a cardiac-related origin.

### 2.4. Statistical Analysis

Continuous variables were reported as means and standard deviations or as medians and ranges. Categorical and nominal variables were reported by prevalence and percentages. Continuous variables between the various study groups were tested for normality using the Shapiro–Wilk test. For abnormal distributions, non-parametric tests were performed by the Mann–Whitney test to compare the two groups. For normal distributions, *t*-tests were performed to compare the two groups. Categorical and nominal variables were analyzed by the Pearson’s chi-square (χ^2^) test. The Kaplan–Meier Survival Analysis procedure and Log Rank (Mantel-Cox) test were performed to test changes over time. A *p* value of <0.05 was considered to be statistically significant. Data were analyzed using SPSS27.

## 3. Results

### 3.1. Patient Characteristics

Between the years 2011 and 2017, 267 patients 70 years old and below (young cohort) and 89 patients 80 years old and above (elderly cohort) underwent a DCB procedure at our institution. Most of the patients were male. It was interesting to see that the percentage of females was much higher in the elderly group (*p* < 0.0001). Both groups had a high prevalence of diabetes mellitus and hyperlipidemia without a significant difference between them (*p* = 0.534 and *p* = 0.946, respectively). For the other baseline characteristics (cigarette smoking, prior known ischemic heart disease (IHD), chronic obstructive pulmonary disease (COPD), renal failure, congestive heart failure, and essential hypertension), significant differences were found and matched analyses were performed in order to overcome these differences (Table 1); we selected subgroups matched head-to-head with 69 patients each. Post-match analysis results were consistent with the pre-matching results for all parameters checked (overall MACE, mortality, etc.). None of the patients had severe valvular disease.

### 3.2. Procedural Data

We used DCB for the treatment of both in-stent re-stenosis (main indication) and de novo (off-label) lesions. In total, 252 patients (70.8% of whole cohort) were treated with DCBs for de novo lesions. A total of 104 patients (29.2% of the whole cohort) were treated with DCBs for in-stent restenosis: 57 patients (64%) in the elderly group and 195 patients (73%) in the young group were treated with DCBs for de novo lesions. Bailout stenting was necessary in 13.5% of the cases for both groups (*p* = 0.99). DCB inflation was performed in the presence of acute coronary syndrome in about one-third of the cases for both groups (*p* = 0.899) (Table 2).

### 3.3. Clinical Outcomes

Statistical analyses was performed for primary outcomes in both matched and un-matched cohorts; as mentioned above, no differences were found.

**Figure 2:**
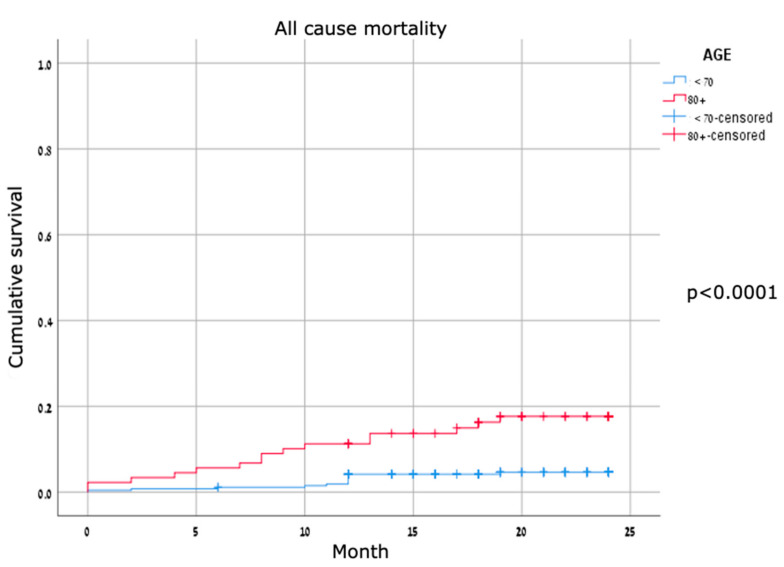
Kaplan–Meier curves for all-cause mortality.

**Figure 3:**
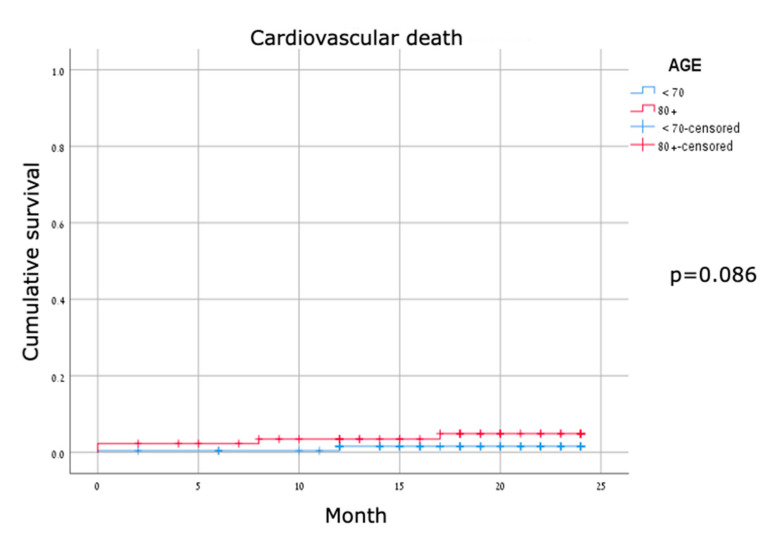
Kaplan–Meier curves for cardiovascular death.

**Figure 4:**
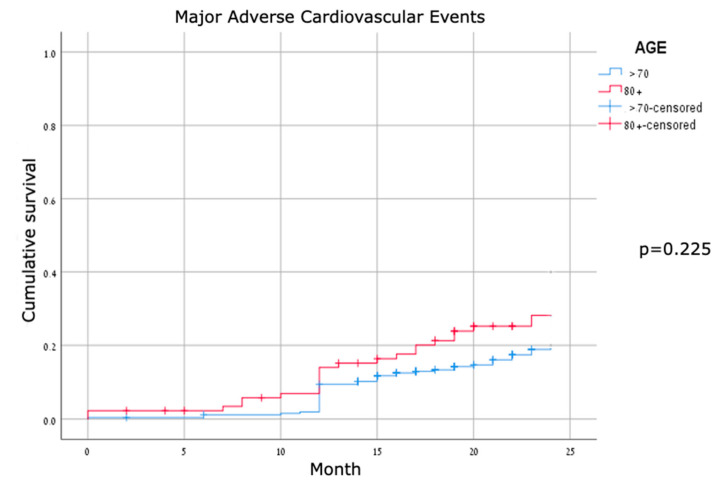
Kaplan–Meier curves for MACE.

Major adverse cardiovascular events were identified in about one-third of the patients in both cohorts, without a significant difference between groups. For MACE compounds—vessel thrombosis, cardiac hospitalizations, target lesion revascularization, and cardiovascular mortality—none were significantly different between the two groups (Table 3). TLR rates were low, consistent with TLR rates published in other studies. Kaplan–Meier survival curves for all-cause mortality showed early separation with significant differences (Figure 2); the curves for cardiovascular mortality were mostly overlapping, and the curves for MACE were mostly parallel and did not show a significant difference between the two age groups (Figure 3 and Figure 4).

## 4. Discussion

To the best of our knowledge, this is the first large-scale study that focuses on a geriatric population regarding the treatment of coronary lesions with drug-coated balloons. Drug-coated balloons have been used more often in the past few years [18] and are becoming accepted as an effective strategy for the treatment of de novo lesions [19], although they have not yet been incorporated into the guidelines for this condition.

The growing proportion of elderly people in the general population and the understanding that special attention should be given to their co-morbidities while tailoring an appropriate treatment strategy give rise to the requirement for the development of a less aggressive treatment approach; the DCB strategy is considered as more amiable methodology for coronary revascularization compared to stent implantation [20,21]; therefore, it may be more suitable for the treatment of the elderly patients in selected scenarios.

The main purpose of this study was to evaluate whether revascularization using a DCB strategy has the same effect on the elderly population as it has on the young population. The recognition of this strategy being as effective and safe in the elderly population as in the younger population can further incline the interventional cardiologist to consider the use of this tactic while treating older patients.

Our analysis showed that geriatric patients treated with DCBs had a higher incidence of MACE, through this was not statistically significant. All-cause mortality was higher in the elderly group, but that is to be expected; cohort matching supported this finding, as old age was the predictor for all-cause mortality. The MACE rates were higher in our study compared to other studies [18,22], which can mostly be attributed to the inclusion of the cardiovascular hospitalization component, which from our point of view is an important aspect concerning the elderly population; if this component was removed, the MACE rates in our study would be complementary to recent data in the literature [23,24]. Special notice should be attributed to TLR, as its occurrence rate in our study is lower than expected and as previously described [25] for both age groups; low TLR for the elderly population means less invasive interventions and less cumulative hospitalization days, which are important prognostic factors for this age group [26,27].

The parameters that were checked regarding the safety, feasibility, and efficacy of the DCB strategy were mostly the same in the elderly population as in the young population; these findings are consistent with smaller-scale studies conducted in the past few years [28,29,30]. Sinaga et al. reported lower TLR, MACE and cardiovascular death rates in a shorter follow-up period (9-months) but without significant differences between the two age groups (below and over 75-years old) as our study did. Mohiaddin et al. mentioned some sub-group analyses in their review showing low bailout stenting rates [31], consistent with our findings as well.

While bleeding risk is considered a major concern and the fact that risk for bleeding increases with age [32,33], the shortest duration of DAPT treatment in the elderly population is advised. Currently, the ESC 2017 DAPT guidelines recommends at least 6-months of DAPT therapy post- DES/DCB deployment in stable CAD and at least 12-months of DAPT therapy in clinical settings of ACS [15]. Shorter durations may apply for HBR [15]. The fact that no foreign body is deployed dramatically reduces the risk for late inflammatory response [34], making the DCB deployment site much less thrombogenic [35]; this fact can serve as a pivot for considering a shorter period of DAPT treatment. Recent studies suggest even a 1 month duration of DAPT therapy post- DCB deployment [36,37].

Selection of an appropriate, effective, and less hazardous coronary revascularization strategy is crucial for the survival of older patients, both peri-procedural and mid- to long-term. In this study, we were able to extrapolate data which support the mid- to long-term survival post- DCB procedure with a comparable low incidence of CV death, while maintaining low TLR in older patients. Our findings are consistent with recently published trial results [19,38], supporting the efficacy and safety profile of this procedure and showing that it is acceptable for implementation in the geriatric population.

### Limitations

Our study is a single center, retrospective study and may not entirely reflect the real-world experience. On the other hand, it maintains low diversity in preparation and deployment techniques.

The lack of intra-coronary imaging (OCT, IVUS) for the investigation of coronary lesions pre-, post- and mid- to long-term after DCB deployment limits us in order to better understand the failure or success of this procedure. Moreover, the size of the elderly cohort is relatively small (and yet, the largest up to date) and may not fully mirror the entire population nation-wide and world-wide. Lastly, no distinction was made between the DCB and uncoated balloons treatments for each cohort.

## 5. Conclusions

The aim of this study was to reflect the mid- to long-term consequences post- DCB procedure in the elderly population. Regarding the results, we found that the use of the DCB strategy for the elderly population can be regarded as safe, effective, and promising. Although having more complex anatomy and comorbidities, the elderly population did not have worse outcomes compared to the younger population. The search for stent-less solutions may lead to an increased usage of DCBs for both de novo and in-stent restenosis lesions. Shorter durations of DAPT therapy should be investigated thoroughly following DCB deployment and, if compliant with the accepted guidelines, can certainly be beneficial to geriatric patients in terms of lowering the bleeding risk and possibly affecting daily medical practice.

## Figures and Tables

**Table 1:** Baseline characteristics.

	Age Groups						Pearson Chi-Square Tests
	<70		80+		Total		
	Count	%	Count	%	Count	%	
Sex (Male)	230	86.10%	55	61.80%	285	80.10%	<0.0001
Smoker	137	51.30%	7	7.90%	144	40.40%	<0.0001
Prior IHD	75	28.1%	39	43.8%	114	32.0%	0.006
COPD	10	3.70%	7	7.90%	17	4.80%	0.114
Renal failure	27	10.10%	22	24.70%	49	13.80%	0.001
DM	107	40.10%	39	43.80%	146	41.00%	0.534
Hyperlipidemia	193	72.30%	64	71.90%	257	72.20%	0.946
CHF	8	3.00%	9	10.10%	17	4.80%	0.006
HTN	162	60.70%	81	92.00%	243	68.50%	<0.0001
2 years survival	245	91.80%	69	77.50%	314	88.20%	<0.0001

IHD—ischemic heart disease, COPD—chronic obstructive pulmonary disease, DM—diabetes mellitus, CHF—congestive heart failure, HTN—hypertension.

**Table 2:** Procedural data.

		Age Groups						Pearson Chi-Square Tests
		<70		80+		Total		
		Count	%	Count	%	Count	%	
Restensis vs. Denovo	Denovo	195	73.00%	57	64.00%	252	70.80%	0.106
	Restensis	72	27.00%	32	36.00%	104	29.20%	
Bailout stenting		36	13.50%	12	13.50%	48	13.50%	0.990
ACS		97	36.30%	33	37.10%	130	36.50%	0.899

ACS—Acute coronary syndrome.

**Table 3:** The 24-month outcomes, all lesion types.

	Age						Log Rank (Mantel-Cox)
	<70		80+		Total		
	Count	%	Count	%	Count	%	
Vessel thrombosis	0	0	0	0	0	0	
Cardiac hospitalizations	93	33.7%	34	38.2%	129	36.2%	0.234
TLR	21	7.9%	6	6.7%	27	7.6%	0.891
Over-all MACE	99	37.1%	37	41.6%	136	38.2%	0.225
Cardiovascular mortality	4	1.5%	4	4.5%	8	2.2%	0.086
All-cause mortality	12	4.5%	15	16.9%	27	7.6%	<0.0001

TLR—Target lesion revascularization.

## Data Availability

The data presented in this study are available on request from the corresponding author.

## References

[1] Kim S., Lee J.-S., Kim Y.-H., Kim J.-S., Lim S.-Y., Kim S.H., Kim M., Ahn J.-C., Song W.-H. (2022). Favorable Vasomotor Function after Drug-Coated Balloon-Only Angioplasty of De Novo Native Coronary Artery Lesions. J. Clin. Med..

[2] Schwartz R.S., Henry T.D. (2002). Pathophysiology of coronary artery restenosis. Rev. Cardiovasc. Med..

[3] Gray W.A., Granada J.F. (2010). Drug-Coated Balloons for the Prevention of Vascular Restenosis. Circulation.

[4] Sella G., Gandelman G., Teodorovich N., Tuvali O., Ayyad O., Abu Khadija H., Haberman D., Poles L., Jonas M., Volodarsky I. (2022). Mid-Term Clinical Outcomes Following Drug-Coated Balloons in Coronary Artery Disease. J. Clin. Med..

[5] Yerasi C., Case B.C., Forrestal B.J., Torguson R., Weintraub W.S., Garcia-Garcia H.M., Waksman R. (2020). Drug-coated balloon for de novo coronary artery disease: JACC state-of-the-art review. J. Am. Coll. Cardiol..

[6] Afilalo J., Karunananthan S., Eisenberg M.J., Alexander K.P., Bergman H. (2009). Role of Frailty in Patients with Cardiovascular Disease. Am. J. Cardiol..

[7] Veronese N., Sigeirsdottir K., Eiriksdottir G., Marques E., Chalhoub D., Phillips C.L., Launer L.J., Maggi S., Gudnason V., Harris T.B. (2017). Frailty and Risk of Cardiovascular Diseases in Older Persons: The Age, Gene/Environment Susceptibility-Reykjavik Study. Rejuvenation Res..

[8] Fernandes J., Gomes C.D.S., Guerra R.O., Pirkle C.M., Vafaei A., Curcio C.-L., de Andrade A.D. (2021). Frailty syndrome and risk of cardiovascular disease: Analysis from the International Mobility in Aging Study. Arch. Gerontol. Geriatr..

[9] Wleklik M., Denfeld Q., Lisiak M., Czapla M., Kałużna-Oleksy M., Uchmanowicz I. (2022). Frailty Syndrome in Older Adults with Cardiovascular Diseases–What Do We Know and What Requires Further Research?. Int. J. Environ. Res. Public Health.

[10] Hilmer S.N., McLachlan A., Le Couteur D. (2007). Clinical pharmacology in the geriatric patient. Fundam. Clin. Pharmacol..

[11] Bell S.P., Orr N.M., Dodson J.A., Rich M.W., Wenger N.K., Blum K., Harold J.G., Tinetti M.E., Maurer M.S., Forman D.E. (2015). What to Expect From the Evolving Field of Geriatric Cardiology. J. Am. Coll. Cardiol..

[12] Goldwater D., Wenger N.K. (2021). Patient-centered care in geriatric cardiology. Trends Cardiovasc. Med..

[13] Alexander K.P., Newby L.K., Cannon C.P., Armstrong P.W., Gibler W.B., Rich M.W., Van de Werf F., White H.D., Weaver W.D., Naylor M.D. (2007). Acute coronary care in the elderly, part I: Non–ST-segment–elevation acute coronary syndromes: A scientific statement for healthcare professionals from the American Heart Association Council on Clinical Cardiology: In collaboration with the Society of Geriatric Cardiology. Circulation.

[14] Merinopoulos I., Gunawardena T., Wickramarachchi U., Ryding A., Eccleshall S., Vassiliou V.S. (2018). Percutaneous coronary intervention in the elderly: Are drug-coated balloons the future?. Curr. Cardiol. Rev..

[15] Valgimigli M., Bueno H., Byrne R., Collet J.-P., Costa F., Jeppsson A., Jüni P., Kastrati A., Kolh P., Mauri L. (2018). 2017 ESC focused update on dual antiplatelet therapy in coronary artery disease developed in collaboration with EACTS. Eur. J. Cardio-Thoracic Surg..

[16] Thygesen K., Alpert J.S., Jaffe A.S., Chaitman B.R., Bax J.J., Morrow D.A., White H.D., Executive Group on behalf of the Joint European Society of Cardiology (ESC)/American College of Cardiology (ACC)/American Heart Association (AHA)/World Heart Federation (WHF) Task Force for the Universal Definition of Myocardial Infarction (2018). Fourth universal definition of myocardial infarction. J. Am. Coll. Cardiol..

[17] Garcia-Garcia H.M., McFadden E.P., Farb A., Mehran R., Stone G.W., Spertus J., Onuma Y., Morel M.-A., Van Es G.-A., Zuckerman B. (2018). Standardized End Point Definitions for Coronary Intervention Trials: The Academic Research Consortium-2 Consensus Document. Circulation.

[18] Elgendy I.Y., Gad M.M., Elgendy A.Y., Mahmoud A., Mahmoud A.N., Cuesta J., Rivero F., Alfonso F. (2020). Clinical and Angiographic Outcomes With Drug-Coated Balloons for De Novo Coronary Lesions: A Meta-Analysis of Randomized Clinical Trials. J. Am. Hear. Assoc..

[19] Merinopoulos I., Gunawardena T., Wickramarachchi U., Richardson P., Maart C., Sreekumar S., Sawh C., Wistow T., Sarev T., Ryding A. (2021). Long-term safety of paclitaxel drug-coated balloon-only angioplasty for de novo coronary artery disease: The SPARTAN DCB study. Clin. Res. Cardiol..

[20] Unverdorben M., Vallbracht C., Cremers B., Heuer H., Hengstenberg C., Maikowski C., Werner G.S., Antoni D., Kleber F.X., Bocksch W. (2015). Paclitaxel-coated balloon catheter versus paclitaxel-coated stent for the treatment of coronary in-stent restenosis: The three-year results of the PEPCAD II ISR study. EuroInterv. J. EuroPCR Collab. Work. Group Interv. Cardiol. Eur. Soc. Cardiol..

[21] Venetsanos D., Lawesson S.S., Panayi G., Tödt T., Berglund U., Swahn E., Alfredsson J. (2018). Long-term efficacy of drug coated balloons compared with new generation drug-eluting stents for the treatment of de novo coronary artery lesions. Catheter. Cardiovasc. Interv..

[22] Megaly M., Rofael M., Saad M., Rezq A., Kohl L.P., Kalra A., Shishehbor M., Soukas P., Abbott J.D., Brilakis E.S. (2019). Outcomes with drug-coated balloons in small-vessel coronary artery disease. Catheter. Cardiovasc. Interv..

[23] Uskela S., Kärkkäinen J.M., Eränen J., Siljander A., Mäntylä P., Mustonen J., Rissanen T.T. (2019). Percutaneous coronary intervention with drug-coated balloon-only strategy in stable coronary artery disease and in acute coronary syndromes: An all-comers registry study. Catheter. Cardiovasc. Interv..

[24] Jeger R.V., Farah A., Ohlow M.-A., Mangner N., Möbius-Winkler S., Leibundgut G., Weilenmann D., Wöhrle J., Richter S., Schreiber M. (2018). Drug-coated balloons for small coronary artery disease (BASKET-SMALL 2): An open-label randomised non-inferiority trial. Lancet.

[25] Liu W., Zhang M., Chen G., Li Z., Wei F. (2020). Drug-Coated Balloon for De Novo Coronary Artery Lesions: A Systematic Review and Trial Sequential Meta-analysis of Randomized Controlled Trials. Cardiovasc. Ther..

[26] Tal S. (2021). Length of hospital stay among oldest-old patients in acute geriatric ward. Arch. Gerontol. Geriatr..

[27] O'Keeffe S., Lavan J. (1997). The Prognostic Significance of Delirium in Older Hospital Patients. J. Am. Geriatr. Soc..

[28] Yu X., Wang X., Zhang W., Liu B., LU D., Sun F., JI F. (2016). Study of efficacy and safety of paclitaxel drug coated balloon in elderly patients with de novo coronary disease. Chin. J. Geriatr..

[29] Miao P., Wu Z., Ren L. (2019). Clinical efficacy of paclitaxel drug-coated balloons in treating acute coronary syndrome in patients with high bleeding risk. Chin. J. Geriatr..

[30] Sinaga D.A., Ho H.H., Zeymer U., Waliszewski M., Jafary F.H., Ooi Y.W., Loh J.K.K., Tan J.K.B., Ong P.J.L. (2015). Drug coated balloon angioplasty in elderly patients with small vessel coronary disease. Ther. Adv. Cardiovasc. Dis..

[31] Mohiaddin H., Wong T.D.F.K., Burke-Gaffney A., Bogle R.G. (2018). Drug-Coated Balloon-Only Percutaneous Coronary Intervention for the Treatment of De Novo Coronary Artery Disease: A Systematic Review. Cardiol. Ther..

[32] Li L., Geraghty O.C., Mehta Z., Rothwell P.M., Study O.V. (2017). Age-specific risks, severity, time course, and outcome of bleeding on long-term antiplatelet treatment after vascular events: A population-based cohort study. Lancet.

[33] Costa F., van Klaveren D., James S., Heg D., Räber L., Feres F., Pilgrim T., Hong M.-K., Kim H.-S., Colombo A. (2017). Derivation and validation of the predicting bleeding complications in patients undergoing stent implantation and subsequent dual antiplatelet therapy (PRECISE-DAPT) score: A pooled analysis of individual-patient datasets from clinical trials. Lancet.

[34] Giannini F., Naim C., Costopoulos C., Latib A., Colombo A. (2013). Drug-coated balloons in interventional cardiology. Expert Rev. Cardiovasc. Ther..

[35] Verdoia M., Negro F., Kedhi E., Suryapranata H., Marcolongo M., De Luca G. (2021). Benefits with drug-coated balloon as compared to a conventional revascularization strategy for the treatment of coronary and non-coronary arterial disease: A comprehensive meta-analysis of 45 randomized trials. Vasc. Pharmacol..

[36] Corballis N.H., Ma M.V.S.V., Vassiliou V., Eccleshall S.C. (2020). Duration of dual antiplatelet therapy in elective drug-coated balloon angioplasty. Catheter. Cardiovasc. Interv..

[37] Rissanen T.T., Uskela S., Eränen J., Mäntylä P., Olli A., Romppanen H., Siljander A., Pietilä M., Minkkinen M.J., Tervo J. (2019). Drug-coated balloon for treatment of de-novo coronary artery lesions in patients with high bleeding risk (DEBUT): A single-blind, randomised, non-inferiority trial. Lancet.

[38] Jeger R.V., Farah A., Ohlow M.-A., Mangner N., Möbius-Winkler S., Weilenmann D., Wöhrle J., Stachel G., Markovic S., Leibundgut G. (2020). Long-term efficacy and safety of drug-coated balloons versus drug-eluting stents for small coronary artery disease (BASKET-SMALL 2): 3-year follow-up of a randomised, non-inferiority trial. Lancet.

